# Enhanced thermoelectric properties in anthracene molecular device with graphene electrodes: the role of phononic thermal conductance

**DOI:** 10.1038/s41598-020-67964-w

**Published:** 2020-07-02

**Authors:** Saeideh Ramezani Akbarabadi, Hamid Rahimpour Soleimani, Zahra Golsanamlou, Maysam Bagheri Tagani

**Affiliations:** Computational Nanophysics Laboratory (CNL), Department of Physics, University of Guilan, Rasht, 41335-1914 Iran

**Keywords:** Chemical physics, Condensed-matter physics

## Abstract

Density functional theory (DFT) and the non-equilibrium Green’s function (NEGF) formalism in the linear response regime were employed to investigate the impact of doping on the electronic and phononic transport properties in an anthracene molecule attached to two metallic zigzag graphene nanoribbons (ZGNRs). Boron (B) and nitrogen (N) atoms were used for doping and co-doping (NB) of carbon atoms located at the edge of the anthracene molecule. Our results show that B doping enhances the electronic transport in comparison with the other dopants which is due to its ability to increase the binding energy of the system. The chemical doping of the anthracene molecule mainly impacts on the thermopower which results in a significantly enhanced electronic contribution of the figure of merit. On the contrary, considering the effect of phononic thermal conductance suppresses the figure of merit. However, by taking into account the effect of both electron and phonon contributions to the thermal conductance, we find that the thermoelectric efficiency can be improved by B doping. The potential role of the phononic thermal conductance in shaping the thermoelectric properties of molecular junctions has been ignored in numerous studies, however, our findings demonstrate its importance for a realistic and accurate estimation of the thermoelectric figure of merit.

## Introduction

Thermoelectric materials provide a great opportunity for direct conversion of heat to electricity and vice versa, that can be employed for a wide range of applications^1–3^. The efficiency of thermoelectric materials is described by the dimensionless figure of merit (*ZT*), that depends on a number of variables: Seebeck coefficient (thermopower, *S*), electric conductance (*G*), electronic thermal conductance ($\kappa _{\mathrm{el}}$), and phononic thermal conductance ($\kappa _{\mathrm{ph}}$), as follows^4^:

1 $$\begin{aligned} ZT = \frac{S^2 T G}{\kappa _{\mathrm{el}} + \kappa _{\mathrm{ph}}}, \end{aligned}$$

where *T* is the absolute temperature. When the phononic thermal transport vanishes, i.e. $\kappa _{\mathrm{ph}} \rightarrow 0$, the total figure of merit (*ZT*) can be roughly approximated by its electronic contribution ($Z_{\mathrm{el}}T$):

2 $$\begin{aligned} Z_{\mathrm{el}}T = \frac{S^2 G}{\kappa _{\mathrm{el}}}\,T = \frac{S^2}{L}, \end{aligned}$$

where $L = \kappa _{\mathrm{el}} / GT$ is the Lorenz number. By introducing $Z_{\mathrm{el}}T$, the total figure of merit can be re-expressed as follows:

3 $$\begin{aligned} ZT = \frac{Z_{\mathrm{el}}T}{1 + (\kappa _{\mathrm{ph}} / \kappa _{\mathrm{el}})}. \end{aligned}$$

where $\kappa _{\mathrm{ph}} / \kappa _{\mathrm{el}}$ is the ratio of the phononic and electronic parts of the thermal conductance which can be used to discuss the effect of phononic thermal conductance on *ZT*. In fact, the interplay between several thermoelectric parameters ultimately shapes the thermoelectric efficiency of the system via the figure of merit given by Eq. (1), which is determined by thermoelectric transport coefficients (see Eqs. (6)–(9) in “Methods” section) in the linear response regime. According to Eq. (1), in order to obtain a higher value for *ZT*, the thermoelectric nanojunction needs to have a greater value of *S* and *G*, along with a small value of the total thermal conductance, i.e. $\kappa = \kappa _{\mathrm{el}} + \kappa _{\mathrm{ph}}$^5^. Therefore, given the diverse repertoire of uninvestigated materials, the search for thermoelectric systems with the desired efficiency can be accelerated by a realistic estimation of thermoelectric variables, specially the thermopower, with a reasonable accuracy.

Among different materials, graphene attracted a lot of interest in recent years due to its exceptional physical, thermal and electrical properties, e.g. high Seebeck coefficient and electric conductance at room temperature^6^. In the past years, a number of experiments showed that graphene nanoribbons (GNRs) can be realized by different physical or chemical methods^7,8^. Unique electronic transport features of graphene hold promise for future nanodevice applications^9,10^, e.g., it can be used to fabricate high performance electronic devices^11^. In particular, zigzag graphene nanoribbons (ZGNRs) can serve as ideal metallic electrodes instead of traditional gold electrodes^11,12^. In fact, covalent contacts may be created when the central scattering region is composed of carbon-based materials^13^.

Recent experimental studies have developed various methods for the atomically precise and chemically stable synthesis of molecular devices with graphene-based electrodes realized by, e.g., mechanically controlled graphene break junctions^14,15^, and chemical deposition of nanoribbons^16–18^, or by feedback-controlled electroburning deposition of molecules inside a few-layer graphene nanogap^19^. In this way, several experiments were able to measure switching^20,21^, charge transport^22^, and thermoelectric properties^23^ in the graphene-based structures. Inspired by these experimental settings, numerous theoretical studies investigated different aspects of thermoelectric properties in graphene-based devices^24–27^. Furthermore, a number of studies explored the impact of different parameters, e.g., coupling geometry^28,29^ or doping process^30–32^ on the thermoelectric properties of graphene-based molecular junctions. In particular, Zheng et al. studied the effect of nitrogen (N) and boron (B) doping on the electron transport in ZGNRs by employing first principle calculations^33^. They found that bound and quasi-bound states may be caused by a BN co-doping, leading to different desirable transport features. Biel et al. introduced a first principle framework of quantum transport in B- and N-doped GNRs, which they found a resonant backscattering and an unconventional acceptor–donor transport in ZGNRs^34^.

Anthracene ($\mathrm{C}_{14}\hbox {H}_{10}$) is a solid tricyclic aromatic hydrocarbon which is made up of three fused benzene rings. Anthracene molecular nanojunctions are suitable candidates for thermoelectric effect studies since they have good conduction properties^35^. Several experimental studies have measured the electron transport properties in structures where a single molecule bridges between two metallic electrodes^36–38^. Theoretical and computational studies have shown that a number of important experimental results can be reproduced by first principle methods, for instance, numerical results on the I-V characteristics of the anthracene-dithiol structure with gold electrodes show quantum behaviors which are consistent with recent experimental results for the anthrylacetylene^39–41^. In particular, alkanethiol-based molecular nanojunctions attracted a lot of attention due to their experimental repeatability^42–44^. Although such systems provide a unique substrate to examine the correspondence between experimental measurements and theoretical calculations, they suffer from poor conducting properties, and therefore, have limited availability in functional devices. However, additional quantum states in the vicinity of the Fermi level may be induced by functional group substitutions^45^. In comparison to pure butanethiol structures, butanethiol junctions substituted with amino show a better thermoelectric performance^46^, so that even a single-molecule refrigerator has been proposed based on them^47^.

Substituting atoms in molecules with another atom is a conventional method to chemically manipulate the properties of molecular devices for cutting-edge applications. In this context, doping B or N atoms is a well-established framework to enhance the thermoelectric features of molecular junctions^30–32^. In fact, the substitutional doping process significantly affects the charge carrier transport in molecular junctions^30–32^. Inspired by experimental situations that has recently been achieved in graphene-based break junction experiments^14^, or by chemical deposition of nanoribbons^17,18^, in this study we investigated the impact of chemical doping on thermoelectric properties of the anthracene molecular junction with graphene electrodes by employing density functional theory (DFT) jointly with the non-equilibrium Green’s function (NEGF) formalism in the linear response regime. Our results show that charge and phonon transports can be regulated chemically by using various dopants that can be categorized as electron-withdrawing or electron-donating. The emergence of strongly localized electronic states can significantly change the transmission of the system depending on the type of the dopant atom. Non-vanishing thermopower significantly enhances the electronic contribution of the figure of merit. However, the effect of the phononic thermal conductance on the thermoelectric properties of molecular junctions has been ignored in numerous studies. Our results indicate that in a wide range of parameters, the magnitude of the phononic thermal conductance is greater than the electronic contribution, in this way, resulting in the suppression of the total figure of merit. Hence, taking into account the effect of the phononic contribution of the thermal conductance is crucial for a realistic and accurate prediction of the figure of merit.

## Methods

**Figure 1:**
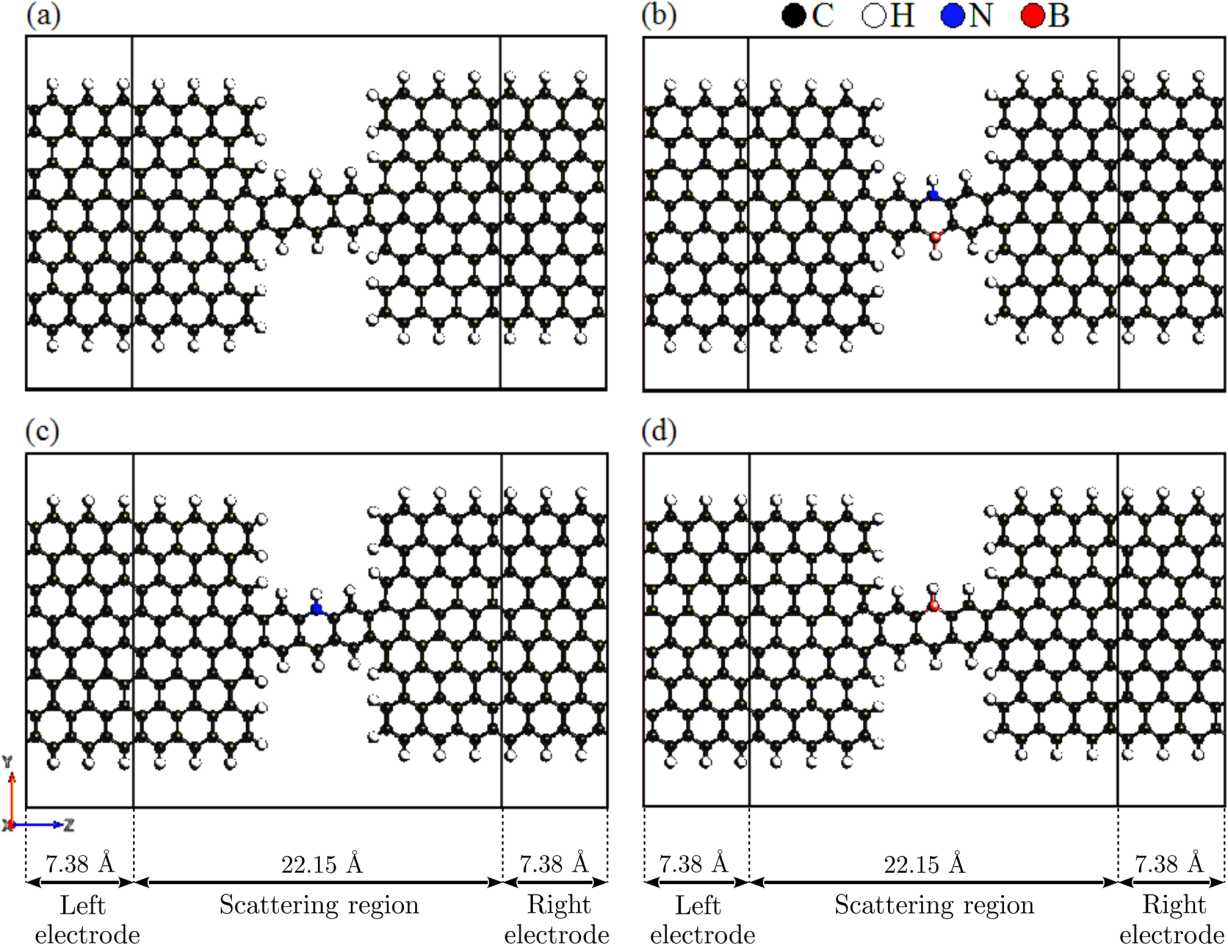
(**a**) The device model of the anthracene molecule sandwiched between ZGNR leads. The edge carbon atoms of the anthracene molecule are substituted by (**b**) NB, (**c**) N, or (**d**) B atoms. Various parts of the sample and their corresponding dimension are indicated at the bottom of the figure.

Molecular structures considered in this study are shown in Fig. 1, that can be characterized by three regions: Left electrode (7.38 Å), scattering region (22.15 Å), and right electrode (7.38 Å) labeled in Fig. 1. In order to establish the doped structures, one of the hydrogen-passivated carbon atoms in the anthracene molecule is replaced by a single B or N atom. In this setting, the anthracene molecule is connected to the metallic ZGNR using a strong covalent bond. The geometry of devices is constructed by optimizing left, right and the central regions of structures shown in Fig. 1. The structures were composed of the non-doped anthracene molecule (see Fig. 1a), and doped with NB, N or B atoms in the scattering region of the anthracene molecule shown in Fig. 1b–d, respectively. Furthermore, we checked the dependence of the results on the width or length of the electrodes, and found that the considered dimensions can produce reliable results.

DFT calculations were performed as implemented in the SIESTA computer program^48^. Transport properties of the system were investigated using the NEGF approach as implemented in the TranSIESTA routine^49^. The electronic structure calculations were carried out via DFT with the Perdew-Burke-Ernzerhof (PBE) generalized gradient approximation (GGA)^50^. The k-point sampling is $1 \times 1 \times 400$ in x, y, and z directions, respectively, where z is the transport direction. The energy cutoff is chosen to be $150\,\mathrm{Ry}$.

Transport properties of the structures are calculated via the Landauer–Buttiker transmission formalism defined in terms of the Green’s function^51^:

4 $$\begin{aligned} T(\varepsilon ) = \mathrm{Tr} [{\mathbf {G}}^{\mathrm {r}}(\varepsilon ) \Gamma _{\mathrm {L}}(\varepsilon ) {\mathbf {G}}^{\mathrm {a}}(\varepsilon ) \Gamma _{\mathrm {R}}(\varepsilon )], \end{aligned}$$

where $\Gamma _{\alpha } = - 2 \, \mathrm {Im} \Sigma _{\alpha }$ is defined by the self-energy $\Sigma _{\alpha }$ of the lead $\alpha$, ${\mathbf {G}}^{\mathrm {r}}(\varepsilon ) = [\varepsilon {\mathbf {S}} - {\mathbf {H}} - \Sigma _{\mathrm {L}}(\varepsilon ) - \Sigma _{\mathrm {R}}(\varepsilon )]^{-1}$ is the retarded Green’s function, and ${\mathbf {G}}^{\mathrm {a}}(\varepsilon ) = [ {\mathbf {G}}^{\mathrm {r}}(\varepsilon )]^{\dagger }$ is the advanced Green’s function, with ${\mathbf {H}}$ and ${\mathbf {S}}$ being the Hamiltonian matrix and the overlap matrix, respectively. Charge (*I*) and heat ($I_{\mathrm{Q}}$) currents calculations were performed by the Keldysh NEGF formalism^51^. Note that the temperature gradient ($\Delta T$), and the voltage difference ($\Delta V$) in the linear response regime are assumed to be small, and therefore, one can expand the charge and heat currents in terms of $\Delta T$ and $\Delta V$ to the first order^51,52^:

5 $$\begin{aligned} \begin{array}{l} I = e^2 L_0 \Delta V + \frac{e}{T} L_1 \Delta T,\\ I_{\mathrm{Q}} = - e L_1 \Delta V - \frac{1}{T} L_2 \Delta T, \end{array} \end{aligned}$$

where $L_n = \hbar ^{-1} \int d \varepsilon (\varepsilon - \mu )^n \, T(\varepsilon ) \, (-\partial f(\varepsilon ) / \partial \varepsilon )$ is the Lorenz function, $f(\varepsilon )$ denotes the equilibrium Fermi–Dirac function, and $\mu$ is the chemical potential.

Thermoelectric coefficients depend on quantities $L_n$, that can be calculated based on the transmission properties of the structure. For instance, the electrical conductance and the electron contribution to the thermal conductance can be written as follows, respectively:

6 $$\begin{aligned} G= & {} e^2 L_0,\end{aligned}$$

7 $$\begin{aligned} \kappa _{\mathrm{el}}= & {} (1 / T) (L_2 - L_1^2 / L_0). \end{aligned}$$

Note that a significant fraction of the thermal conductance value comes from the electronic contribution, however, in order to take into account the effect of the phononic contribution, $\kappa _{\mathrm{ph}}$ is also considered in this study. Thermopower (Seebeck coefficient) can be written as the ratio of the induced voltage difference to the applied temperature difference when the current vanishes:

8 $$\begin{aligned} S = - \frac{\Delta V}{\Delta T} = - (1 / eT)(L_1 / L_0), \end{aligned}$$

then, $\kappa _{\mathrm{ph}}$ can be calculated from classical methods. The phononic thermal conductance can be obtained from the phonon transmission function $T_{\mathrm{ph}}(\omega )$ via the Landauer-type formula^53^:

9 $$\begin{aligned} \kappa _{\mathrm {ph}} = \frac{\hbar ^2}{2\pi } \int _{0}^{\infty } d\omega \, T_{\mathrm {ph}}(\omega ) \left( \frac{\partial f_{\mathrm {ph}}(\omega )}{\partial \omega }\right) , \end{aligned}$$

where $f_{\mathrm{ph}}(\omega )$ is the Bose–Einstein distribution function for heat carriers. Phonons are characterized by the classical dynamical matrix of the system. The ReaxFF potential is used for the parameterization of the potential. The phonon transmission function and the frequency of phonons ($\omega$) in the elastic transport regime can be written in terms of NEGF for the scattering region:

10 $$\begin{aligned} T_{\mathrm {ph}}(\omega ) = \mathrm {Tr} [ {\Lambda }_{\mathrm {R}}(\omega ) {{\mathbf {D}}}(\omega ) {\Lambda }_{\mathrm {L}} (\omega ) {{\mathbf {D}}}^{\dagger } (\omega ) ]. \end{aligned}$$

The phonon retarded Green’s function can then be calculated in a similar way by substituting ${\mathbf {H}}\rightarrow {{\mathbf {K}}}$, $\varepsilon {{\mathbf {S}}}\rightarrow \omega ^2{{\mathbf {M}}}$ and ${{\Sigma }}_{\mathrm {L,R}}\rightarrow {\Pi }_{\mathrm {L,R}}$:

11 $$\begin{aligned} {{\mathbf {D}}}(\omega ) = [ \omega ^2 {{\mathbf {M}}} - {{\mathbf {K}}} - {{\Pi }}_{\mathrm {L}}(\omega ) - {{\Pi }}_{\mathrm {R}}(\omega ) ]^{-1}, \end{aligned}$$

where ${\mathbf {K}}$ denotes the force constant matrix, ${\mathbf {M}}$ is a diagonal matrix consists of the atomic masses, ${{\Pi }}_{\mathrm {L,R}}$ represents the self-energies, and ${{\Lambda }}_{\mathrm {L,R}}(\omega )=i[{\Pi }_{\mathrm {L,R}}(\omega ) -{\Pi }^{\dagger }_{\mathrm {L,R}}(\omega )]$.

## Results

The edge atoms of ZGNR are hydrogen-terminated in order to achieve more stable physical properties and to saturate the carbon 2*p* edge states that can result in localized states in the pure GNR^54^. We considered a single carbon substitution by B and N, and double substitution by NB atoms at the edge of the sample (see Fig. 1b–d). Both N and B atoms are the most commonly used dopants for carbon-based materials^55,56^. In the studied device geometry shown in Fig. 1, the size of the scattering region is 22.15 Å and the size of the ZGNR electrodes is 7.38 Å. Within this range of parameters, the effect of phonon-phonon scattering can be ignored since the size of the scattering region or the width of the ZGNR electrodes is much smaller than the mean free path of graphene ($l \simeq 677 \, \mathrm {nm}$) at room temperature^26^.

**Figure 2:**
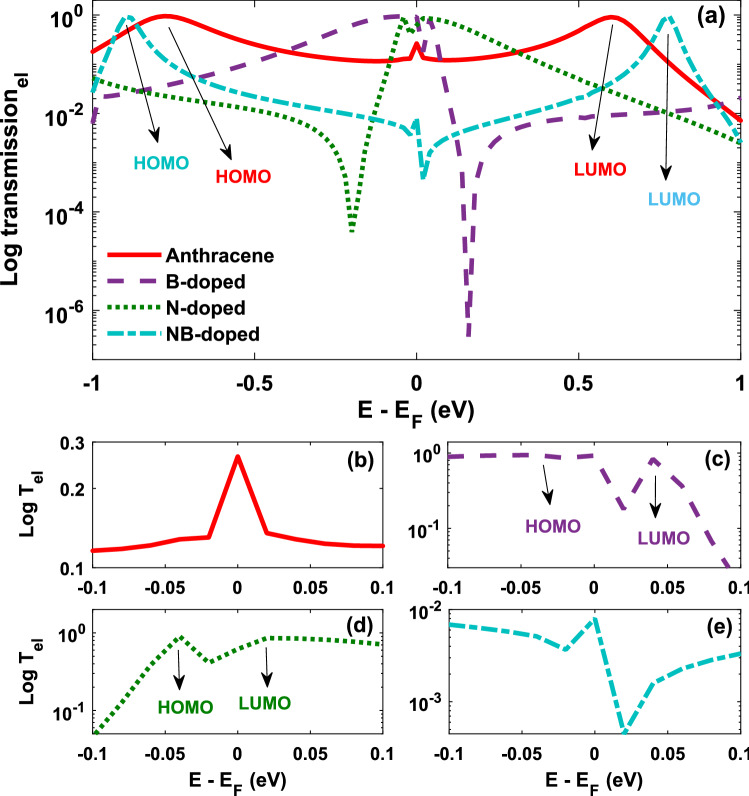
Logarithm of the transmission coefficient of the anthracene molecule sandwiched between ZGNR leads versus energy in the non-doped and doped (with B, N or NB atoms) molecular junctions. Logarithm of the transmission coefficient is plotted with a higher resolution for the (**b**) non-doped, (**c**) B-doped, (**d**) N-doped and (**e**) NB-doped molecular junctions. The arrows and labels indicate molecular orbitals (HOMO or LUMO) contributing to the transmission coefficient.

Figure 2 shows the logarithm of transmission coefficient of the non-doped and doped anthracene molecules coupled to the ZGNR electrodes versus energy. The position of molecular energy levels of the molecular junction in the transmission spectrum (peaks that are marked in Fig. 2) may shift due to constructive/destructive quantum interference of electronic waves in the molecule in the presence of dopants in comparison with the molecular energy levels of the non-doped junction (marked for anthracene in Fig. 2a). In particular, B doping shifts the highest occupied molecular orbital (HOMO) towards the Fermi level (marked in Fig. 2c), whereas in the N-doped molecule, the lowest unoccupied molecular orbital (LUMO) is shifted towards the Fermi level (marked in Fig. 2d). Furthermore, when the molecular junction is doped with NB atoms, the HOMO-LUMO gap is increased in comparison to the non-doped molecule (see Fig. 2a), whereas it is decreased for the B- and N-doped molecules. The peaks appeared in the transmission spectrum (marked in Fig. 2) are related to the energy eigenvalues of the structure. Hence, one can hypothesize that the conductance spectrum can reveal the electronic structure of the molecule. When carbon atoms are replaced by B, N or NB atoms, the electronic characteristics can be modified. The shift of the HOMO-LUMO gap can be described by the effect of p-type and n-type impurities on the B-, N- or NB-doped anthracene molecule^30^. The electron-donating nature of B doping increases the energy of $\pi$ electron system because HOMO-LUMO gap shifts to the right side of the Fermi energy and the HOMO energy is close to the Fermi energy ($E_{\mathrm{F}}$) (p-type transport)^57^. In contrast, for electron-withdrawing N doping, electron transport occurs through LUMO (n-type transport) and the HOMO-LUMO gap shifts to the left side of the Fermi energy (see Fig. 2), which is in accordance with previous experimental and theoretical results^54,58^.

Furthermore, Fig. 2 shows that when the molecular junction is doped, not only the position of the molecular orbitals (as argued in the previous paragraph), but also the magnitude of the transmission coefficient at the Fermi energy may shift in comparison with the transmission coefficient of the non-doped configuration. This can be attributed to the quantum interference among electronic waves passing through the molecular rings which ultimately leads to the energetic separation of the HOMO or LUMO levels from the Fermi level (based on the type of the dopant atom). By adding B and N atoms, the magnitude of transmission in the Fermi energy increases. These changes are a direct consequence of doping. In this range of energy, B- and N-doped molecules do not yield any charge localization, and hence, a greater electron transmission is obtained (see Fig. 4)^32^. The reduction of the transmission coefficient at the Fermi energy in NB doping can be addressed by considering a destructive quantum interference effect of the transmission near the Fermi energy and the presence of two dopants which reduce the symmetry of the system^59^. By increasing the interaction effect due to the incorporation of further impurities, the peak of the transmission coefficient is reduced compared to the other molecular junctions. In the case of NB-doped molecule, electronic states are positioned in the vicinity of the electrodes. These charge localizations at nanoscale are shown to suppress the probability of electron transport (see Fig. 2)^32^. Since N doping is considerably more electronegative than B doping, the donation of electron density into the electrode is smaller for the N-doped molecular junction, providing less stabilization of N–C bond. Thus, the binding energy for the B-doped molecular junction is stronger than the N-doped one^60^, and the trend in binding energies for our studied atoms is N < B. The broadening resulted in the energy levels shown in Fig. 2 is a consequence of the molecule-electrode coupling.

**Figure 3:**
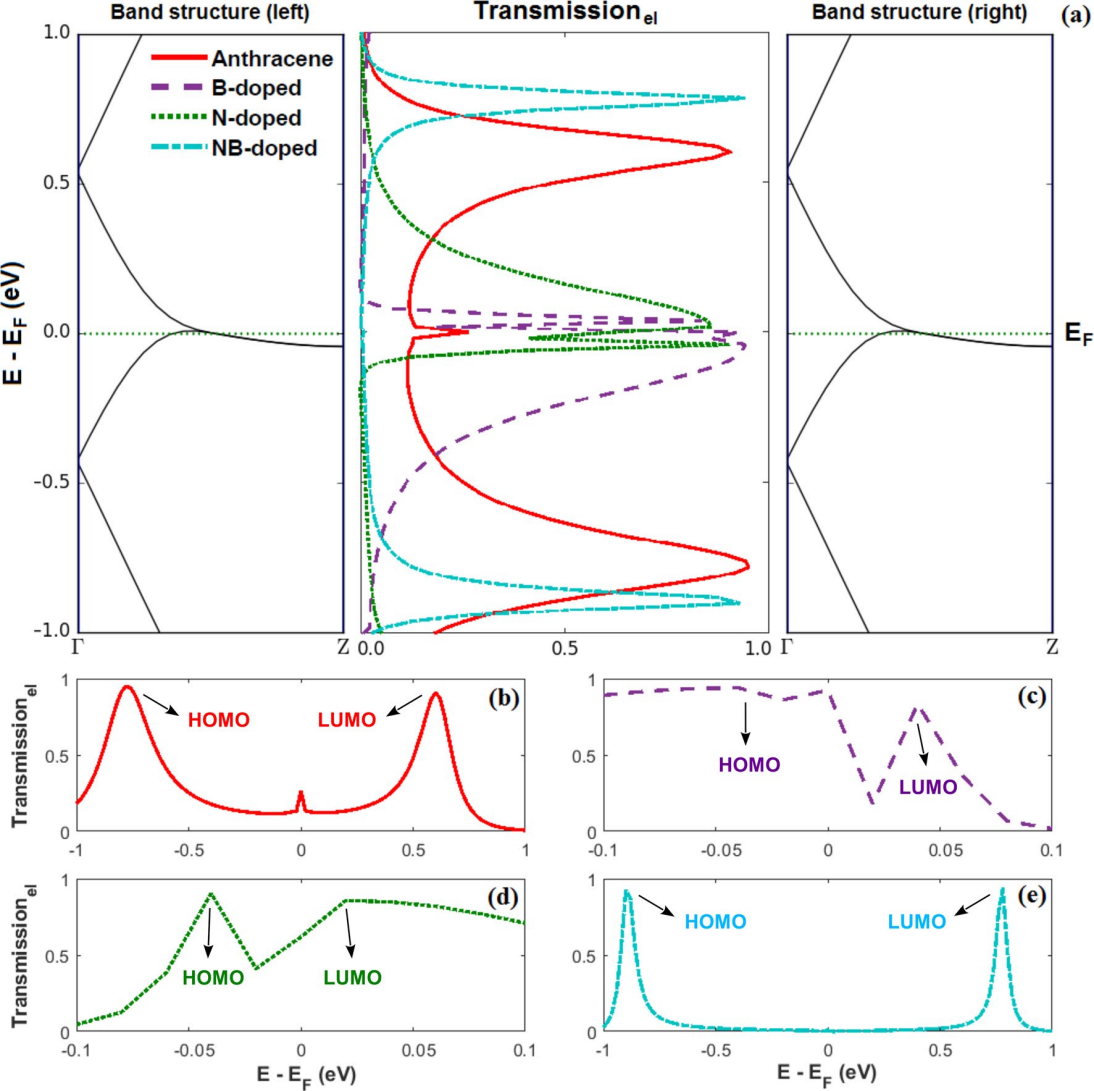
(**a**) Band structure of the left (left) and right electrodes (right), and the transmission coefficient of the non-doped and doped (with B, N or NB atoms) anthracene molecule sandwiched between ZGNR leads versus energy (middle). The transmission coefficient is shown separately (with a higher resolution) for the (**b**) non-doped, (**c**) B-doped, (**d**) N-doped and (**e**) NB-doped molecular junctions. The arrows and labels indicate molecular orbitals (HOMO or LUMO) contributing to the transmission coefficient.

To further clarify the above phenomena, we analysed the band structure of the electrodes (see Fig. 3). The bands of the two electrodes have identical structures where this constructive similarity is the prerequisite of ideal transmission channels. Figure 3 shows that the electrodes are metals since they do not have a band gap. The analysis of the band structure (energy gap) of low-layer graphene does not show any gap. This structure becomes metal progressively with an increase in the number of layers. The band structure of electrodes and the transmission coefficient in Fig. 3 shows that the band gap of the NB-doped molecular junction is considerably increased in comparison to the other configurations (cf. Fig. 3b, c, e) since no states have been appeared between HOMO and LUMO peaks in this case. One can find that the position of molecular levels of the studied systems also depends on the type of dopants. For instance, in the p-type B doping, HOMO gets closer to the Fermi level, but the n-type N doping increases the Fermi energy. This procedure can be attributed to the electrostatic screening effect of p-type and n-type impurities.

**Figure 4:**
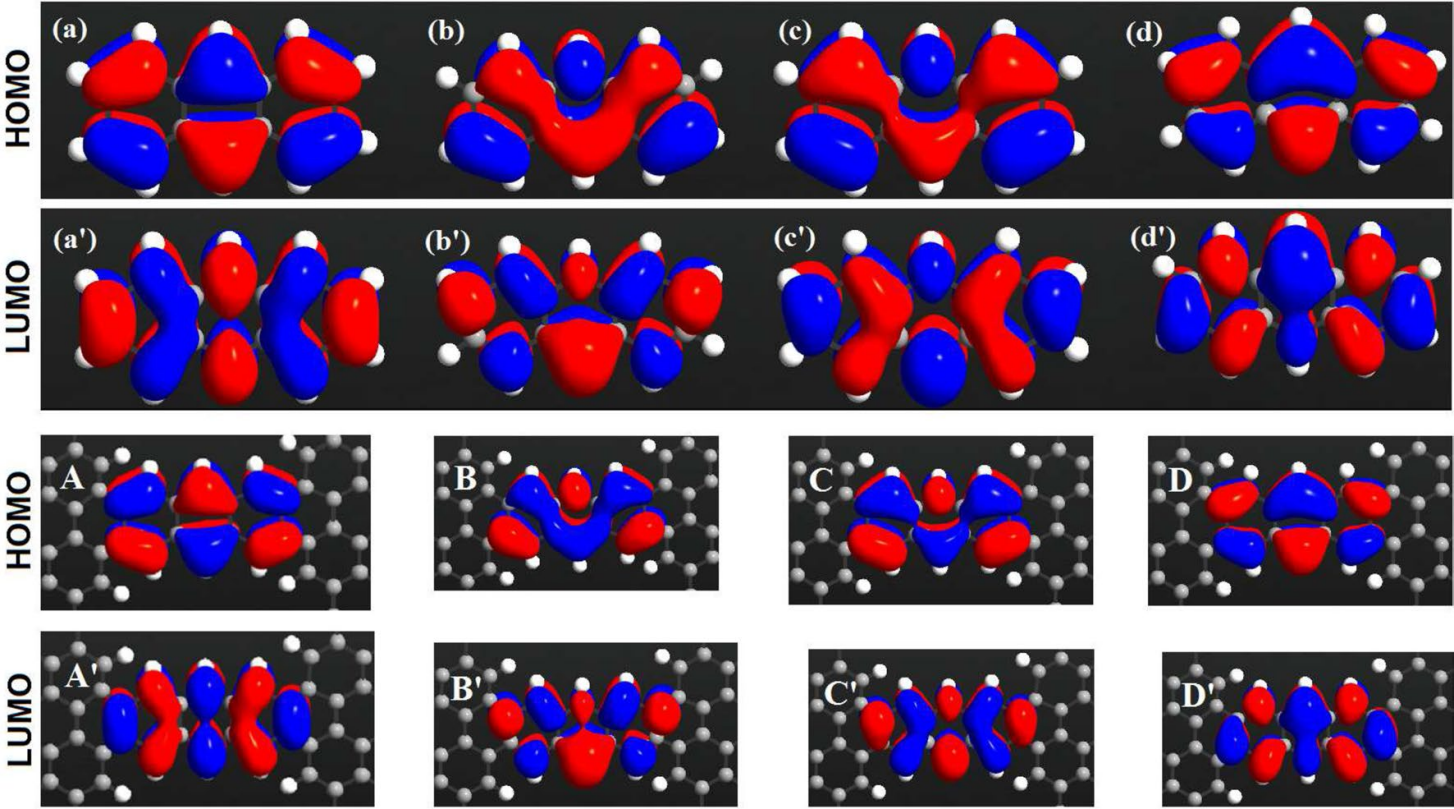
(Top) Calculated HOMO and LUMO orbitals of the ($\mathbf{a,a}^\prime$) non-doped, ($\mathbf{b,b}^\prime$) NB-doped, ($\mathbf{c,c }^{\prime }$) N-doped, and ($\mathbf{d,d }^\prime$) B-doped anthracene. (Bottom) The isosurface diagrams of the projected self-consistent Hamiltonian eigenstates for the ($\mathbf{a,a}^\prime$) non-doped, ($\mathbf{B,B }^\prime$) NB-doped, ($\mathbf{C,C }^\prime$) N-doped, and ($\mathbf{D,D }^\prime$) B-doped anthracene. The isovalue of each molecular orbital surface is $0.05 \, \mathrm{a.u}$.

The HOMO and LUMO analysis were employed to determine the charge transfer through the molecule. The investigation of the wave function of the system reveals that the electron absorption is related to a transition from ground state to the first excited state. This is generally explained by a single electron excitation from HOMO to LUMO. The HOMO and LUMO energies are very important terms in quantum chemistry. Both HOMO and LUMO are the dominant orbitals participating in chemical processes. The HOMO energy denotes the capability of electron donating of the system, whereas the LUMO energy characterizes the capability of electron accepting. This can be verified by the transmission coefficient and the HOMO and LUMO analysis shown in Figs. 3 and 4.

The calculated HOMO and LUMO are shown in Fig. 4. To understand the origin of the modifications of the transmission caused by the doping process, the projected self-consistent Hamiltonian (PSH) eigenstates of the system are calculated. All the states are anti-symmetric in the plane of the anthracene molecule. This means that they represent $\pi$ orbitals of the anthracene molecule, i.e. they are linear combinations of $\pi$ orbitals on each carbon atom which are perpendicular to the plane of the molecule. Figure 4 shows that HOMOs are localized on the donor anthracene (B atom) moiety. Conversely, LUMOs are positioned over the acceptor due to the non-bonding lone pair of N atom^61^. This is illustrated by the transmission coefficient in Fig. 3. Therefore, these arguments illustrate that the HOMO energy is reduced as the electron-donating properties of the donor parts are increased. We showed that the anthracene molecule manipulated by B atom has a lower HOMO energy level than those molecules doped by the other dopants. This effect is seen in push-pull systems where the HOMO energy is determined by the donor strength^62^. Figure 4d has the greatest value of conductance since HOMO is the closest energy level to $E_{\mathrm{F}}$, whereas the lowest value of conductance belongs to Fig. 4c′ which its LUMO is the furthest energy level from $E_{\mathrm{F}}$. This can be verified by the transmission coefficient shown in Fig. 2.

**Figure 5:**
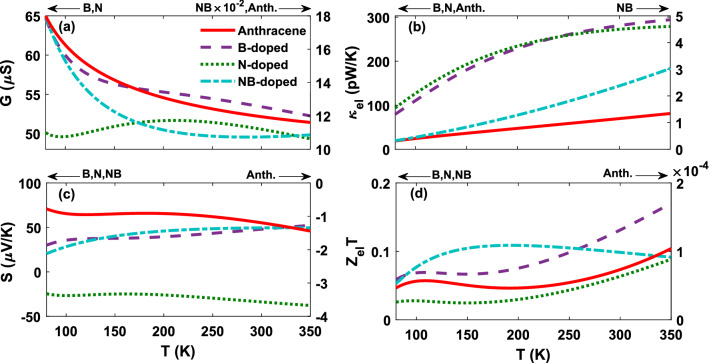
Temperature dependence of thermoelectric transport coefficients: (**a**) electrical conductance, (**b**) electron thermal conductance, and (**c**) thermopower. (**d**) Electronic contribution of the figure of merit in the anthracene molecule sandwiched between ZGNR leads in the non-doped and doped (with B, N or NB atoms) molecular junctions. The arrows and labels above each panel indicate the y-axis (left or right) in which the thermoelectric coefficients are plotted for each molecular junction.

Figure 5a–c shows the temperature dependency of thermoelectric transport coefficients. The electrical conductance for all molecular junctions is decreased by increasing the temperature except the N-doped configuration (see Fig. 5a). The reason for this descending trend of the electrical conductance is that the Fermi derivative ($- \partial f / \partial \varepsilon$) associated with $L_0$ in Eq. (6) gets broadened around $E_F$ and its overlap with the peaks of the transmission coefficient is decreased as the temperature increases. This ultimately decreases the number of carriers participating in transport resulting in a smaller *G*^63^. In the N-doped molecular junction, however, the Fermi derivative also gets broadened around $E_F$ with temperature increase, but in this case, the Fermi derivative covers additional peaks of the transmission coefficient. Therefore, the number of carriers participating in transport is increased which results in a greater *G* till $T \sim 200 \, \mathrm{K}$, but at higher temperatures *G* is reduced due to the same argument used earlier in the case of other configurations (see Fig. 5a). The highest value of electrical conductance in Fig. 5a corresponds to the B-doped molecular junction due to the enhanced transmission coefficient near the Fermi energy in comparison to the other configurations (see Fig. 2a). The probability of electron transport to the molecular levels is more pronounced in this case. The lowest value of the electrical conductance, however, corresponds to the NB-doped molecular junction which can be attributed to the reduction of the transmission coefficient near the Fermi energy (see Fig. 2a) and the increase of the HOMO-LUMO gap in comparison to the other configurations (illustrated in Fig. 3e).

We investigated the electron thermal conductance behavior with temperature. The electron thermal conductance in Eq. (7) is associated with two terms: $L_2$ and $L_1^2 / L_0$. It has been shown that in the context of thermoelectric studies on the doped molecular junctions, the contribution of $L_2$ to the electron thermal conductance is more than $L_1^2 / L_0$^63^. Therefore, when analysing the behavior of the electron thermal conductance, we focused on the behavior of $L_2$. In order to support this argument, both the $L_2$ and $L_1^2 / L_0$ terms are sketched versus energy for all considered molecular configurations in the Supplementary Information (see Supplementary Fig. S1), which illustrates that the contribution of the $L_2$ term to the electron thermal conductance is one order of magnitude greater than the $L_1^2 / L_0$ term. In the relation of $L_2$ given by the Lorenz function, only the term that changes with temperature is the Fermi derivative, and hence, increasing the temperature makes more areas of the transmission peak to be enclosed by the Fermi derivative peak and the number of carriers taking part in transport increases. According to the transmission coefficient in Fig. 2, the electron thermal conductance increases with temperature. The distance between the peak of the transmission coefficient and the Fermi level is notable for NB doping, therefore, the electron thermal conductance is small. The electron thermal conductance shown in Fig. 5b increases with the enhancement of temperature since electrons and holes carry more thermal energy in this case. This increase is uniform for the non-doped anthracene molecular junction, but in the presence of dopants, its ascending trend is relatively nonlinear. The greatest value of the electron thermal conductance belongs to the N-doped molecular junction up to $T \sim 250 \, {\mathrm{K}}$, which then follows an ascending trend with increasing the temperature (see Fig. 5b). This is because the transmission coefficient peak in this case is slightly closer to the Fermi energy in comparison to the other dopants, and therefore, the number of carriers in transport increases.

Figure 5c shows the magnitude of the thermopower as a function of temperature. One of the key factors to enhance the thermoelectric efficiency of molecular structures is taking advantage of materials with high thermopower. These materials are typically marked by a sharp peak in the transmission function close to the Fermi level. This can be explained by the approximated expression for the thermopower, $S \varpropto \textit{d}\varepsilon \log (\textit{T}(\varepsilon ))$, i.e. the slope of the transmission is a function of energy on the logarithmic scale^54^. One of the applications of the sign of the thermopower is to determine the transport mechanism or relative position of the Fermi energy^64^: A positive sign indicates a p-type conduction, meaning that the Fermi energy lies near the HOMO level^65^. The sign of thermopower is positive for B and NB doping, whereas the N-doped and non-doped anthracene molecular junctions are characterized by a negative thermopower. This can be attributed to the electron-donating and electron-accepting nature of B and N atoms, respectively.

Figure 5c also shows that the maximum value of thermopower for the NB-doped molecule is the greatest value among the molecular junctions. This can be attributed to the fact that on the one hand, the difference between the Fermi energy and the peak of the transmission coefficient is increased in this case (see Fig. 3e), and on the other hand, as shown in Fig. 2e, the NB-doped molecule is characterized by a sharp slope of the transmission coefficient with respect to energy at $E-E_F = 0$. This leads to a greater overlapping between the transmission coefficient and the Fermi derivative near $E_F$, and ultimately, the value of thermopower is increased. In the case of the B- and N-doped molecule, the distance of energy levels from the Fermi energy is decreased in comparison to other configurations (see Fig. 3c, d), leading to a mild slope of the transmission coefficient near the Fermi energy (see Fig. 2c, d), but $L_0$ or *G* is increased (see Fig. 5a). Therefore, the ratio $L_1 / L_0$ becomes smaller in Eq. (8), and the thermopower decreases consequently^66^. The lowest value of the thermopower belongs to the non-doped anthracene. The reason is that the overlapping between the transmission coefficient and the Fermi derivative is small near the Fermi energy in comparison to the other configurations due to the mild slope of the transmission coefficient with respect to energy at $E-E_F = 0$ (see Fig. 2b). However, in the case of B, N and NB doping, the value of *S* is increased in comparison to the non-doped molecule.

**Figure 6:**
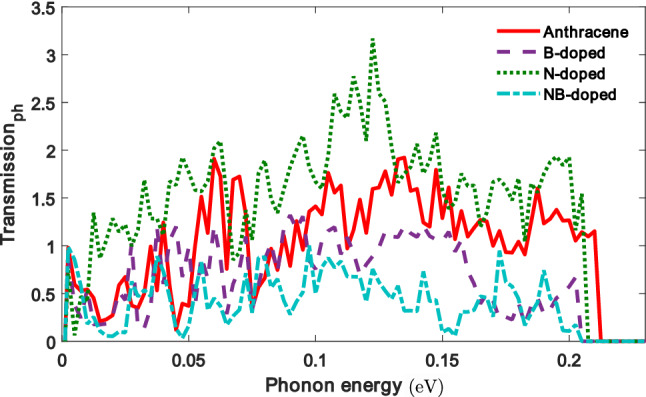
The phonon transmission coefficient of the anthracene molecule sandwiched between ZGNR leads versus phonon energy in the non-doped and doped (with B, N or NB atoms) molecular junctions.

Ultimately, the effect of all thermoelectric transport coefficients modifies the electronic contribution of the figure of merit (shown in Fig. 5d). Since $Z_{\mathrm{el}}T$ quadratically depends on *S*, it practically follows the behavior of the thermopower from Fig. 5c. According to Fig. 5d, up to $T \sim 250 \, \mathrm{K}$, the maximum of $Z_{\mathrm{el}}T$ belongs to the NB-doped molecular junction, however, at higher temperatures, the B-doped molecular junction has the greatest value of the $Z_{\mathrm{el}}T$. The lowest value of $Z_{\mathrm{el}}T$ belongs to the non-doped anthracene molecular junction since it has a smaller *S*. In recent years, with the advent of nanotechnology the value of the figure of merit has been improved in different nanostructures, e.g., Garcia-Suarez et al. obtained $Z_{\mathrm{el}}T \simeq 0.02$ for conjugated oligo-based (phenylene-ethynylenes) molecular junctions at $T = 350 \, {\mathrm{K}}$^67^, and Branislav et al. calculated $Z_{\mathrm{el}}T \simeq 0.09$ for ZGNR-C10-ZGNR junction at $T = 350 \, \mathrm{K}$^26^. In the present study, B-doped molecular junction shows the greatest value of the electronic figure of merit, i.e. $Z_{\mathrm{el}}T \simeq 0.17$ at $T = 350 \, \mathrm{K}$, which is significantly improved in the presence of the dopants.

The effect of phononic contribution to the thermal conductance has been ignored in numerous studies by taking advantage of approximated approaches^63,65,68^. However, taking into account the effect of the phononic thermal conductance is necessary for a more realistic estimation of the figure of merit. Hence, we also considered the effect of the phononic thermal conductance which is determined by using classical methods. To estimate the phonon contribution, the phonon transmission coefficient of the molecular junctions is calculated (shown in Fig. 6). The comparison of the phonon transmission between the molecular junctions clearly indicates a deviation from the non-doped molecule. Doping with B and NB atoms shifts the phonon transmission peaks of the system towards lower frequencies in comparison to the peaks of the non-doped and N-doped anthracene molecules where the amplitude of peaks is also decreased. The phonon transmission in this case also forms additional peaks besides the peaks seen in the non-doped molecular junction. Since in the doped molecules there are more transmission routes for these peaks, the quantum interference effect between localized states and nearby extended states results in multiple resonant peaks in the phonon transmission^13^.

**Figure 7:**
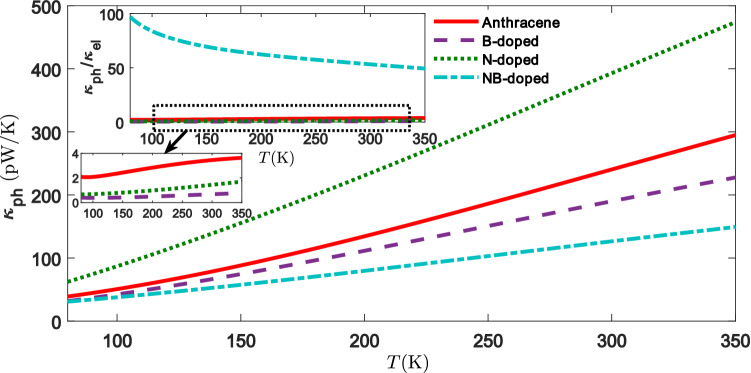
Temperature dependence of the phononic thermal conductance. The inset shows the ratio of phonon and electron thermal conductances ($\kappa _{\mathrm{ph}} / \kappa _{\mathrm{el}}$).

**Figure 8:**
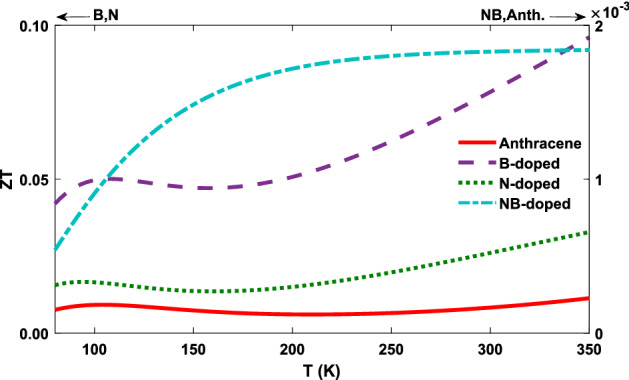
Temperature dependence of the total figure of merit for the B-doped, N-doped (left y-axis), NB-doped and non-doped (right y-axis) molecular junctions which consists of both the electronic and phononic linear response transport coefficients.

The temperature dependence of the phononic thermal conductance is illustrated in Fig. 7, where the expected ballistic behavior is obtained with increasing the temperature. The phononic thermal conductance increases by the temperature enhancement for all of the molecular junctions. As shown in Fig. 7, the magnitude of the phononic thermal conductance for the N, NB-doped and non-doped molecules is greater than the electronic thermal conductance (inset to Fig. 7). Thermoelectric materials characterized with large values of *G* are often associated with significant values of $\kappa _{\mathrm{el}}$, which is due to the similar behavior relevant to the transmission function. When $\kappa _{\mathrm{el}} > \kappa _{\mathrm{ph}}$, the effects of *G* and $\kappa _{\mathrm{el}}$ are counteracted by each other, and therefore, the improvement of *ZT* is rather a challenging task in this case^5^. The behavior of the thermal conductances (see Figs.5b, 7) shows that *ZT* (shown in Fig. 8) is much suppressed when $\kappa _{\mathrm{el}} < \kappa _{\mathrm{ph}}$^69^. Note that for the N, NB-doped and non-doped molecular structures the magnitude of $\kappa _{\mathrm{ph}}$ is greater than $\kappa _{\mathrm{el}}$ (see inset to Fig. 7). The phononic thermal conductance increases for N-doped molecular junction because of an increase of the phonon transmission in comparison to the other molecular junctions.

The ratio $\kappa _{\mathrm{ph}} / \kappa _{\mathrm{el}}$ is one of the most essential factors in determining the value of *ZT* which is inversely proportional to *ZT* (see Eq. (3)). According to Eq. (3), *ZT* is strongly suppressed for the NB-doped molecular junction in comparison to the other structures since $\kappa _{\mathrm{ph}} / \kappa _{\mathrm{el}} > 10$ in the range $100 \, {\mathrm{K}}< T < 350 \, \mathrm{K}$ (see Fig. 8)^67^. The figure of merit for the B-doped molecular junction in Fig. 8 is more than the other structures since it has the lowest dependency on the phononic thermal conductance, i.e. $\kappa _{\mathrm{ph}} / \kappa _{\mathrm{el}}$ is smaller for this junction. The greatest value of *ZT* was obtained for the B-doped molecular junction with $ZT \simeq 0.10$ at $T = 350 \, \mathrm{K}$. The greatest value of *ZT* at room temperature was also calculated for the B-doped molecular junction with $ZT \simeq 0.08$, which is significantly improved in the presence of dopants. This illustrates that for all of the molecular junctions $\kappa _{\mathrm{ph}} / \kappa _{\mathrm{el}} > 1$, and therefore, $ZT < Z_{\mathrm{el}}T$^67^. In order to overcome such a suppression, efficient strategies should be implemented for decreasing the role of parasitic phonons.

## Discussion

In this study, we employed the NEGF-DFT framework in the linear response regime to investigate the electron and phonon transport in nanoscale thermoelectric devices. We focused on the thermoelectric properties of single-molecule nanojunctions and performed a study of the effect of substitutional impurities on the transport properties. The nanojunctions considered here were composed of an anthracene molecule connected to metallic ZGNR electrodes. We used B, N and NB atoms to dope the carbon atoms located at the edge of the anthracene molecule sample.

Our results show that the doping process significantly impacts on the thermoelectric coefficients of the molecular junction including the electric conductance (Fig. 5a), thermal conductance (Figs. 5b, 7), and thermopower (Fig. 5c). Electron-donating dopants (B and NB) regulate the thermopower value so that it shows a positive sign, whereas the electron-withdrawing group (N) yields a negative value for the thermopower. The greatest improvement of the absolute value of the thermopower was found for the NB-doped molecular junction, however, it has the lowest value of *ZT*. It should be noted that according to Eq. (3), $ZT = Z_{\mathrm{el}}T / (1 + \kappa _{\mathrm{ph}} / \kappa _{\mathrm{el}})$, the ratio of the phononic and electronic parts of the thermal conductance, $\kappa _{\mathrm{ph}} / \kappa _{\mathrm{el}}$, is a key factor in determining the value of *ZT* in the molecular junction. Accordingly, here we showed that the value of *ZT* was strongly decreased in comparison to the other molecular structures, when $\kappa _{\mathrm{ph}} / \kappa _{\mathrm{el}} > 10$ in the range $100 \, {\mathrm{K}}< T < 350 \, \mathrm{K}$. The non-vanishing thermopower significantly increases the electronic contribution of the thermoelectric figure of merit. However, the phononic thermal conductance is, even at room temperature, greater than the electronic contribution, resulting in a significant reduction of the total *ZT* for N, NB-doped and non-doped molecular junctions. Therefore, based on the results presented here, one can conclude that it is essential to consider the phononic thermal conductance for low-conducting molecular nanojunctions to achieve an accurate and reliable estimation of *ZT*.

The electronic contribution of the figure of merit in different nanostructures has been improved by a number of similar studies in recent years. For instance, Garcia-Suarez et al. achieved $Z_{\mathrm{el}}T \simeq 0.02$ for conjugated oligo-based molecular junctions at $T = 350 \, \mathrm{K}$^67^, and Branislav et al. obtained $Z_{\mathrm{el}}T \simeq 0.09$ for ZGNR-C10-ZGNR junction at $T = 350 \, \mathrm{K}$^26^. Here, however, we showed that the greatest value of the electronic figure of merit corresponds to the B-doped molecular junction, i.e. $Z_{\mathrm{el}}T \simeq 0.17$ at $T = 350 \, \mathrm{K}$ which is greatly enhanced in comparison to the aforementioned studies. A number of previous studies, on the other hand, were able to obtain greater values for *ZT* by only considering the contribution of the electronic thermal conductance (i.e. the contribution of the phononic thermal conductance was ignored) or by employing different methods and materials. For example, Golsanamlou et al. improved the thermoelectric efficiency of polyaniline molecular junctions by doping process and reported $ZT \simeq 0.18$ at room temperature^63^. Garcia-Suarez et al. reported $Z_{\mathrm{el}}T \simeq 0.3$ at $T = 350 \, \mathrm{K}$ achieved by redox control of thermopower and figure of merit in cross-conjugated oligo-based (phenylene-ethynylenes) molecular junctions^67^. Here, however, by taking into account the effect of the phononic thermal conductance, our aim was to provide a more realistic estimation of figure of merit in comparison to the other similar studies. We showed that the greatest value of *ZT* belongs to the B-doped molecular junction with $ZT \simeq 0.10$ at $T = 350 \, \mathrm{K}$, whereas, as mentioned earlier, the greatest electronic figure of merit also corresponds to the B-doped molecular junction with $Z_{\mathrm{el}}T \simeq 0.17$ at $T = 350 \, \mathrm{K}$. This reduction in the value of *ZT* with respect to $Z_{\mathrm{el}}T$ clearly illustrates the suppressing effect of the phononic thermal conductance on *ZT* which further highlights the necessity to account for its crucial role in future studies.

B doping is considerably less electronegative than the other dopants (the binding energy of B doping is considerably greater than the other dopants), and hence, the donation of electron density into the electrodes is more significant in this case. This provides more stabilization of the B-C bond, which in turn, increases the thermoelectric efficiency of the system. In fact, different dopants can result in significant variations in the transmission coefficient, mainly due to the alteration of the alignment of the frontier molecular orbital levels and the Fermi energy level^70^. Furthermore, due to the trend of the binding energy, the electrical conductance of the B-doped molecular junction is more than the other considered molecular junctions^60^. In this way, the possibility of regulating molecule’s transport mechanism to be either p- or n-type merely by replacing the dopants is strongly encouraging, since this rather simple idea can be employed in other $\pi$ conducting systems.

From a methods standpoint, GGA calculations are computationally cheaper and faster than similar methods, but they encounter several shortcomings, e.g. they may underestimate orbital shape and energies, band structures and chemical bonds of the system which can impose several limitations on the model used in this study. Frameworks such as local density approximation (LDA)^71^, on the other hand, provide a realistic description of the atomic structure, elastic and vibrational features for a wide range of systems. Shortcomings of LDA makes it not reliable enough to calculate energy considerations of different molecular conformations. However, GGA methods have overcame such pitfalls to a large extent, providing e.g., a more realistic account of energy barriers and adsorption energies for molecules on metal or semiconductor surfaces. On the other hand, the results of several applications suggest that GGA functionals are still too limited to yield a fully consistent improvement over LDA and to describe accurate binding energies^72–74^. Hence, the choice of the most efficient and suitable functional that fits one’s particular problem is still under debate.

Ultimately, the results presented in this study highlight the crucial role of phonon engineering in restricting the thermal conductance of thermoelectric systems, specially in low dimensional materials. By taking the advantage of nanoscale structures such as ZGNR electrodes in this study, our findings indicate that besides the chemical tuning of the electronic transport properties in the molecular system, restricting the phononic contribution of the thermal current may be an efficient approach to improve the value of *ZT*. In break junctions or scanning tunneling microscope (STM) experiments, the alignment of the molecule with leads is not symmetric and also leads are not symmetric. Asymmetry in the coupling between molecule and electrodes dominates the electron and phonon transport. Especially, phonon transport is strongly dependent on the geometry of the coupling, so an asymmetric coupling can increase phonon mismatch in the boundaries, and as a result, significant reduction in phonon thermal transport is observed. In addition, electron transport is also dependent on the geometry of coupling, so electrical conductance can be also affected. Thermopower is usually robust against coupling geometry or even increases by asymmetry, so one could expect that the asymmetric coupling geometry increases figure of merit^58,68^. Hence, findings of theoretical and computational studies on the enhancement of the thermoelectric properties in different nanodevices, and in particular their *ZT*, can provide practical insights for the experimental realization of efficient nanostructures used in a wide range of thermoelectric applications.

## Supplementary information

Supplementary material 1 (pdf 120 KB)

## Data Availability

All data generated or analysed during this study are included in this published article and its Supplementary Information files.
