# Supplementary material for: Enhanced thermoelectric properties in anthracene molecular device with graphene electrodes: the role of phononic thermal conductance
Source: Sci Rep. 2020 Jul 2;10:10922. doi: 10.1038/s41598-020-67964-w (PMC7331582; doi:10.1038/s41598-020-67964-w)
Supplement: Supplementary file 1 — Supplementary material 1 (pdf 120 KB) [file 41598_2020_67964_MOESM1_ESM.pdf]

Supplementary Information for: Enhanced thermoelectric properties in anthracene molecular device with graphene electrodes: The role of phononic thermal conductance

S. Ramezani Akbarabadi[1,*], H. Rahimpour Soleimani[1], Z. Golsanamlou[1], and M. Bagheri Tagani[1]

[1]**Computational Nanophysics Laboratory (CNL), Department of Physics, University of Guilan, Rasht, 41335-1914, Iran**

[*]**sramezani@phd.guilan.ac.ir**

Electron thermal conductance

According to the equation describing the electron thermal conductance, $\kappa_{\rm el} = (1/T)(L_2 - L_1^2/L_0)$, the trade-off between the L_2 and L_1^2/L_0 terms may impact on the value of the electron thermal conductance. Similar thermoelectric studies on the doped molecular junctions have considered the L_2 term only due to its greater contibution to the electron thermal conductance [1]. Furthermore, in order to justify our argument regarding the greater contibution of the L_2 term to the electron thermal conductance in comparison to L_1^2/L_0, as shown in Fig. **S1**, both terms are plotted for the non-doped and doped (with B, N or NB atoms) anthracene molecule sandwiched between ZGNR leads versus energy. Fig. **S1**a shows that in this framework, the value of the L_2 term for considered configurations is one order of magnitude greater than the value of the L_1^2/L_0 term (see Fig. **S1**b). Hence, in this context, we focused on the behavior of the L_2 term when analysing the behavior of the electron thermal conductance.

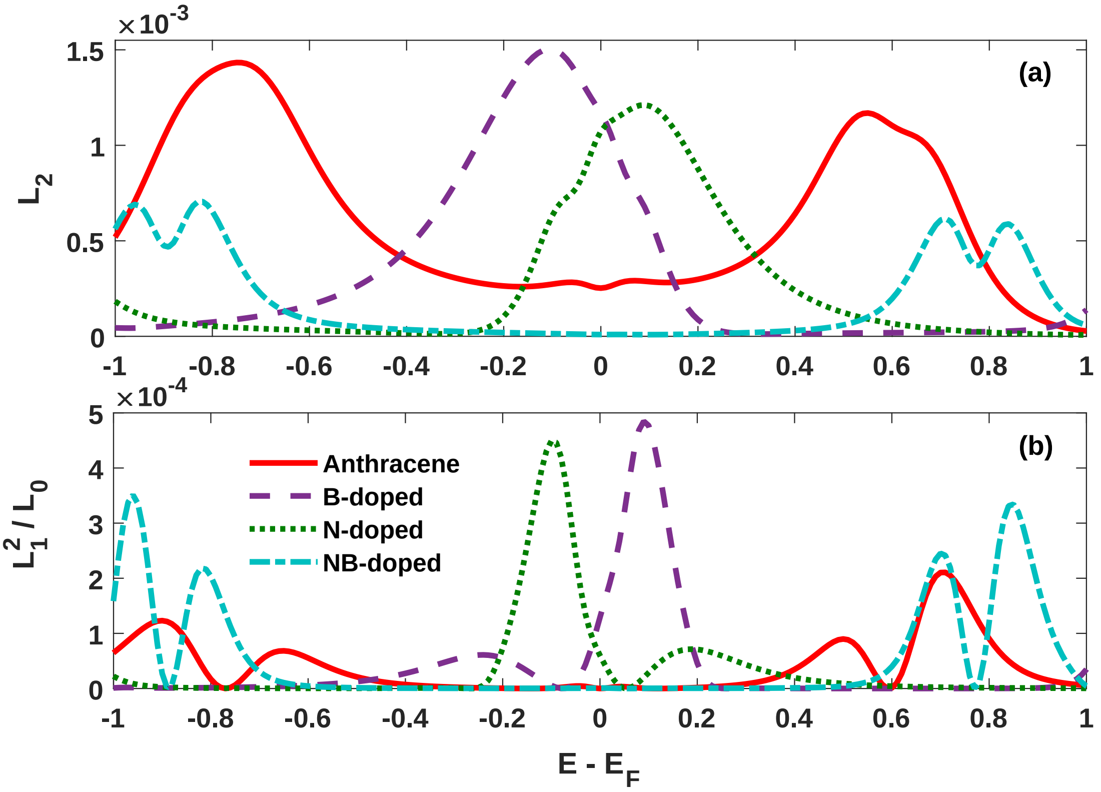

Figure S1. Lorenz functions contributing to the electron thermal conductance. (**a**) L_2 and (**b**) L_1^2/L_0 terms are shown for the non-doped and doped (with B, N or NB atoms) anthracene molecule sandwiched between ZGNR leads versus energy at $T = 350\,\mathrm{K}$.

References

[1] Golsanamlou, Z., Bagheri Tagani, M. & Rahimpour Soleimani, H. Improvement of thermoelectric efficiency of the polyaniline molecular junction by the doping process. *Phys. Chem. Chem. Phys.* **17**, 13466–13471 (2015).
